# Attitudes of Pakistani and Pakistani heritage medical students regarding professionalism at a medical college in Karachi, Pakistan

**DOI:** 10.1186/1756-0500-7-150

**Published:** 2014-03-15

**Authors:** Saima Akhund, Zulfiqar Ali Shaikh, Syed Arif Ali

**Affiliations:** 1 Department of Community Medicine, Dow International Medical College, Dow University of Health Sciences, Karachi, Pakistan; 2 Department of Research, Dow International Medical College, Dow University of Health Sciences, Karachi, Pakistan

**Keywords:** Professionalism, Attributes, Medical education, Undergraduate medical students, Pakistan

## Abstract

**Background:**

An increased interest in professionalism has been reported in the field of medical education due to concerns regarding deterioration of humanism and professional values in the teaching and practice of medicine. The primary aim of this study was to assess attitudes of Pakistani and Pakistani heritage students at a medical college in Pakistan about important elements of professionalism that an ideal medical doctor should possess. A further objective of the study was to determine students’ preferred ways of learning professionalism.

**Methods:**

A written survey was distributed to undergraduate medical students at a public sector medical college at Karachi, Pakistan in 2011. Using the Penn State College of Medicine (PSCOM) Professionalism Questionnaire, attitudes of medical students of semester 1, 5, and 8 regarding professionalism were assessed anonymously.

**Results:**

The mean age of the students was 21.11 ± 2.72 years. Forty-three percent of the respondents were male. Forty percent of the students held Pakistani citizenship. Thirty-five percent students were US citizens with Pakistani parents and twenty-five percent were Pakistani heritage students that had dual citizenships. No significant differences in the elements of professionalism (Accountability, Altruism, Duty, Excellence, Honesty & Integrity and Respect) mean scores or in the overall mean score of professionalism among the various classes were found. The total overall Cronbach alpha value for all elements of the professionalism in the selected classes was above 0.9. The most preferred methods for learning professionalism were role modeling by faculty, case based scenarios and role plays.

**Conclusion:**

The students rated all the attributes of professionalism as important and there was no difference across the study years. The overall internal consistency of each element of professionalism was high in different classes. Faculty role models, case based scenarios and role plays may be used to teach professionalism. As a great majority of students were having a Pakistani heritage rather than complete Pakistani born and bred background, hence findings of the survey may not be taken as representative of typical Pakistani medical students.

## Background

Professionalism has been regarded as a selection of core competencies of physicians since Hippocrates’ time [1,2]. In recent decades, medical profession has increasingly been criticized for erosion of professional and ethical values [3,4]. The reasons for decline in the aforementioned core competencies of physicians include changes in health care delivery patterns, technological advances, and challenging initiatives such as quality assurance and evidence-based medicine [5-8]. The medical community has responded to this criticism by an increased interest in conserving, advancing, researching, teaching, and evaluating professionalism in both education and practice areas [2].

The word “Professionalism” is derived from the Latin *professio,* or public declaration [9]. Though originally referring to open declaration of a religious belief, the term is used to characterize the values, behaviours and attributes professed by individuals involved in the practice of a specific field [2,10]. Several organizations engaged with physician education and training such as the American Board of Internal Medicine, the European Federation of Internal Medicine, the Accreditation Council for Graduate Medical Education, and the American Association of Medical Colleges have listed those essential elements of Professionalism which an individual physician must possess. Despite some variation, the common attributes defined by various organizations include altruism, respect, compassion, integrity, accountability and commitment etc.; building on the foundation principles of patient welfare, autonomy and social justice [11-14].

It has now become the current emphasis of medical educationists that professionalism must be taught explicitly during undergraduate medical studies [3,15,16]. There is a substantial amount of scientific literature that proposes how to formally teach professionalism during preclinical as well as clinical years at medical schools [2,14,17,18].

A number of studies have also provided examples of how different medical schools have incorporated professionalism in their curriculum which includes teaching professionalism as a longitudinal theme throughout the duration of the medical curriculum, teaching humanistic elements of professionalism by specially designing an elective course or by utilizing existing subjects such as Gross Anatomy, and involving local stake holders to suggest professional competencies and hence connect to cultural contexts [8,19-21].

Assessment research to identify what effect such efforts have on student and resident practice have found promising results. A study from the Netherlands on intensive care fellows identified the importance of both formal and informal methods for learning professionalism with the need to expand formal methods [22]. A medical school in Ireland received outstanding professional results from second year medical students who were taught professionalism in an interdisciplinary style [23]. Similarly teaching biomedical ethics as an integrated medical curriculum was found helpful in providing medical students professional insights [24].

Research has indicated that an evaluation of students’ existing perceptions toward professionalism is critical for the development of courses aimed at improving professional behavior [25]. However, professionalism is still not taught explicitly in medical colleges and medical universities in Pakistan and therefore the need for formal inclusion of a curriculum on professionalism in both pre and post graduate medical education in Pakistan has been highlighted [26]. To the best of our knowledge, this is the first study from Pakistan describing perceptions of students regarding professionalism. While the primary aim of this study was to assess attitudes of Pakistani and Pakistani heritage students at a medical college in Pakistan about important elements of professionalism that an ideal medical doctor should possess, a further objective of the study was to determine students’ preferred ways of learning professionalism.

## Methods

A written survey was distributed to undergraduate medical students at a public sector medical college in Karachi, Pakistan in the year 2011. The duration of the Bachelor of Medicine and Bachelor of Surgery (MBBS) course in Pakistan is five years. We contacted semester 1, 5 and 8 medical students to have representation of both pre-clinical and clinical years. The students were approached at the beginning of lecture and were explained about the study objectives and that the details of study and informed consent related information were given at the beginning of the study questionnaire. Students were free to fill out the questionnaires anonymously or return them without answering. At the end of the lecture, questionnaires were distributed to the students. One hundred and twenty seven students answered the survey questionnaire. The Penn State College of Medicine (PSCOM) Professionalism Questionnaire, which has been developed in Pennsylvania USA, was used after obtaining permission for its use from the author [25]. We decided to use this questionnaire because assessment of professionalism is relatively a new area in the field of medical education and only a few of the available instruments for its assessment are validated. Students at the study site were not taught professionalism explicitly. We inquired their current perceptions on the topic with the help of PSCOM Professionalism Questionnaire. This questionnaire had statements on elements of professionalism and students were given a five optioned rating scale of importance to judge the extent of their view of professional values. Numerous studies have used this strategy of provision of some references to students while trying to gauge their view-point on professionalism [27-29].

We did some slight modifications in the demographic section of the PSCOM Professionalism Questionnaire. The demographic questions now include each student’s study year, gender, age, and citizenship. For assessing main elements of a professional behaviour i.e. Accountability, Altruism, Duty, Excellence, Honesty & Integrity and Respect, six randomized sets of statements were provided. The students were asked to rate the degree to which each statement corresponded to their definition of professionalism on a five-point Likert scale (1 = never, 2 = little,3 = some, 4 = much, and 5 = great deal). For example, for assessing the element “Honesty & Integrity”, a total of six statements were distributed in random manner throughout the different sets of statements. One of these statements is as follows: an ideal medical doctor *“Upholds scientific standards and bases decisions on scientific evidence and experience”.* The students were required to choose from the above-mentioned Likert scale options how important that element of professionalism was to them. The maximum score for the statement was 5, for the whole set of statements related to the honesty and integrity element it was 30 and the maximum overall score for all professionalism related elements was one 180. To achieve the second objective of the survey, an additional question regarding preferred method for study of professionalism was added to the survey questionnaire. The options provided with this question were chosen from the published literature on professionalism teaching. The survey was pilot-tested on the undergraduate medical students of a different public sector medical college (n = 5) in Karachi. As a result of pilot testing the further rank ordering of the relative importance of items contained in the original PSCOM Professionalism Questionnaire was removed as students found it difficult and time consuming.

### Setting

Dow International Medical College is very similar to other public sector medical colleges of Karachi in terms of curriculum and examination pattern but the difference is that the majority of its students are children of those Pakistanis who left Pakistan for studies or employment and then have settled abroad. The students therefore present not only Pakistani Muslim upbringing but also share the lifestyle, educational foundation and attitudes typical of developed societies. Their fee payment mode is also international.

### Ethical considerations

The objectives of the study were explained to the students and their informed consent was taken. The study questionnaire was anonymous hence students’ identities were undisclosed. According to the Ethical Research Committee guidelines by the National Bioethics Committee of Pakistan, educational research involving human subjects where no identifying information is collected that can link subjects to the data and where informed consent is taken could be exempt from research ethics committee approval [30].

### Data analysis

The data were analyzed using SPSS 17.0. Simple descriptive statistics (frequencies and percentages) were computed for each categorical variable. Mean and standard deviations were calculated for continuous variables and professionalism elements (Accountability, Altruism, Duty, Excellence, Honesty & Integrity and Respect). To estimate the internal consistency of the elements of professionalism and overall professionalism score, we used Cronbach alpha values. For comparisons between semesters included in our survey (1st, 5th and 8th), we used Analysis of variances (ANOVA).

## Results

There was a total of 193 students enrolled in the selected study semesters. Out of 193 students, 135 were present in their respective classes at the time of data collection and 94% of them responded. Table 1 describes the demographic characteristics of the survey respondents. The student’s mean age was 21.11 ± 2.72 years. Forty-three percent of the respondents were male. Almost 89% of the respondents had completed twelve years of education or had completed their A levels prior to their admission in the medical college whereas 11% had completed a bachelors degree. Before joining the medical college, 44% of the students were residing in USA, 24% in Pakistan, 18% in the Middle East and the rest in Canada. Forty percent of the students had Pakistani citizenship, 35% were US and Canadian citizens and 25% held dual citizenships.

**Table 1:** Demographic characteristics of the respondents from semester 1, 5 and 8 at an international medical college from Karachi, Pakistan

**Variable**	**Total sample**		**(Semester 8)**		**(Semester 5)**		**(Semester 1)**	
	**n = 127**		**n = 25**		**n = 60**		**n = 42**	
**Gender**	n	%	n	%	n	%	n	%
Male	55	43.3	11	20	27	49.1	17	30.9
Female	72	56.7	14	19.4	33	45.8	25	34.7
**Prior degree**								
HSc/FSc/A levels	107	89.1	23	92.0	56	93.3	28	22.8
B.S	13	10.8	2	8.0	3	5%	8	61.5
**Citizenship**								
Pakistani	51	40.2	16	64	20	33.33	15	35.71
USA	34	26.8	6	24	21	35	7	16.67
Canada	9	7.1	1	4	4	6.66	4	9.52
Other	1	0.8	0	0	1	1.66	0	0
Dual	32	25.2	2	8	14	23.33	16	38.09
**Age (Mean ± SD)**	21.11 ± 2.72		23.16 ± 1.99		20.84 ± 1.38		20.29 ± 3.80	

n = 127.

Internal consistency reliability estimates for each element of professionalism included in the PSCOM Professionalism Questionnaire administered to the three classes are given in Table 2. Among the three classes, the reliability estimates are almost the same for all the elements of the professionalism and the total overall Cronbach alpha value was above 0.9. Moreover, none of the reliability estimates in our study were in the lower range.

**Table 2:** Cronbach alpha values of elements of professionalism for the study semesters 1, 5 and 8 students at an international medical college from Karachi, Pakistan

**Element**	**Total sample**	**Semester 8**	**Semester 5**	**Semester 1**
	**n = 127**	**n = 25**	**n = 60**	**n = 42**
Accountability	0.75	0.72	0.72	0.80
Altruism	0.78	0.82	0.72	0.81
Duty	0.72	0.76	0.61	0.81
Excellence	0.75	0.78	0.62	0.83
Honesty & integrity	0.82	0.85	0.74	0.86
Respect	0.82	0.87	0.74	0.87
Overall	0.95	0.96	0.93	0.97

n = 127.

A comparison of the mean (standard deviation) scores for the PSCOM Professionalism Questionnaire for the study semesters 1, 5 and 8 are presented in Table 3. Using ANOVA, no significant differences were found in the elements of professionalism scores or in the overall score among various classes.

**Table 3:** Comparison of elements of professionalism scores (Mean/SD)for the study semesters 1, 5 and students at an international medical college from Karachi, Pakistan

**Element**	**Semester 8**	**Semester 5**	**Semester 1**	**F- test statistic**	P-value*
	**n = 25**	**n = 60**	**n = 42**		
Accountability	22.83(4.41)	23.50(3.66)	23.78(4.42)	0.412	0.66
Altruism	22.12(5.37)	23.23(3.85)	23.17(4.42)	0.610	0.50
Duty	22.95(4.10)	21.75(3.57)	23.10(4.17)	1.67	0.19
Excellence	22.00(4.68)	22.63(3.46)	23.41(4.75)	0.917	0.40
Honesty & integrity	23.20(5.09)	23.07(3.91)	24.15(4.67)	0.708	0.49
Respect	23.83(4.66)	23.78(3.79)	24.65(4.79)	0.533	0.58
Overall	140.09(23.92)	138.55(18.82)	142.45(25.22)	0.319	0.72

n = 127.

*ANOVA test p-value.

Table 4 shows the most preferred method for study of professionalism by the respondents. Thirty-four percent of the respondents preferred learning professionalism as is exhibited by faculty members and 7% preferred learning it from senior colleague. Another 32% of the students wanted to learn professionalism through case-based scenarios.

**Table 4:** Preferred methods for learning professionalism among the study semesters 5 and 8 students at an international medical college from Karachi, Pakistan

**Teaching method**	**n**	**%**
Role modeling by faculty	25	33.8
Role modeling by senior colleagues	5	6.8
Lectures	1	1.4
Reading	3	4.1
Web based course	4	5.4
Case based scenarios	24	32.4
Mentor program	3	4.1
Role play	9	12.2

n = 74.

## Discussion

This study describes the attitudes of medical students toward professionalism at one medical college in Pakistan using the PSCOM Professionalism Questionnaire. The study also highlights approaches that have been found valuable for learning professionalism by the students.

Prior work that had used the American Board of Internal Medicine foundational elements of professionalism for assessing student opinion has found that all student groups agree upon these elements [31,32]. The finding of no significant difference among the professionalism related elemental or overall score among our survey classes could partly be explained due to the fact that in Pakistan no formal activity or course aimed at enhancing professionalism is carried out in undergraduate medical curriculum. Professionalism rather is considered as an attribute that is implicitly learnt by students during their medical school and hospital training years. A study that had followed students over their course of pharmacy training had identified an increase in professionalism scores in later years. This increase was probably due to the inclusion of several professionalism related curricular and co-curricular activities in the course over time [33]. Since this was the first ever study to measure professionalism scores in Pakistan, no comparison values were available. The results of this survey can be used as a base line for assessing time trends or after any intervention regarding inclusion of professionalism in the undergraduate medical curriculum.

One of the strengths of our survey is the composition of our student sample, which includes Pakistani born and bred students as well as students who have had their high school education in the USA, Canada, and other countries. Our findings indicate that irrespective of place of high school education, all Pakistani and Pakistani heritage students have rated aspects of professional behavior as equally important for a medical doctor. There is currently a wealth of evidence from the west that highlights the importance of professionalism from the students’ viewpoint [34-36].

The need for medical schools and teaching hospitals to work continuously on developing and maintaining a high degree of professionalism has been stressed by other studies [37,38]. One of the strategies to inculcate professional ethics among students is to consider their own view on how best this should be taught [34]. In our study, the majority of students have considered role modeling as the most preferred method for learning professionalism. Role modeling by teachers or senior colleagues is considered as an extremely powerful instrument for passing on professional values among medical students as well as residency trainees [39-41]. Some other researchers have argued that the traditional role modeling technique is no longer sufficient, and hence other approaches for teaching professionalism may also be considered such as teaching cognitive-based structured sessions for experiential learning, having formal evaluations, and faculty development activities [15,42].

The other most preferred methods for learning professionalism in our survey were the use of case-based scenarios and role-plays. Use of case-based scenarios for undergraduate medical teaching of professionalism has been advocated by other studies as well. These scenarios were found to improve students’ skills in dealing with ethical issues [43,44]. A study by Ratanawongsa et al. [45] however has found that role-plays and standardized patient or video taped patient-physician interactions as the least-used and the least preferred methods for teaching professionalism by residents. This difference in findings may be due to the use of a different group as undergraduate students and residents may differ in their clinical exposure and judgment.

### Limitations

Limitations of the study include collection of data at only one medical institute. The opinion of students towards professionalism expressed in this study may not typically represent the view point of Pakistani students as 60 percent of the students though had Pakistani heritage but received their basic education from the US and Canadian systems. Secondly, as with other cross-sectional studies, onetime assessment of attitude does not confirm that these attitudes would translate into behaviors. The low number of students in semester 8 as compared to other survey semesters can also be considered a study limitation but since it was the senior most semester of the medical school at the time of study we considered including the viewpoint of its students as important. Moreover, the total enrollment was in semester 8 was 27 and we received responses from 25 of them. The response rate for the question on most preferred method to study professionalism is 74 (Table 4) as this question was added after data was collected from one semester.

## Conclusion

The students rated all the attributes of professionalism (Accountability, Altruism, Duty, Excellence, Honesty & Integrity and Respect) as important and there was no difference across the study years. Faculty role models, case-based scenarios and role-plays may be used to teach professionalism. We propose that the study should be replicated at other medical colleges of Pakistan so as to gain further insights to the views on professionalism held by a larger population of students.

## Competing interest

The authors declare that they have no competing interest.

## Authors’ contribution

SA conceptualized and designed the study, collected the data, reviewed the literature and drafted the manuscript. ZAS participated in designing the study, collected the data, and reviewed the manuscript. SAA performed statistical analysis and contributed in its interpretation. All authors read and approved the final manuscript.
